# Sleep Maintenance Insomnia in Older Adults: Cardiometabolic Comorbidities and Evidence of Antiviral Pathways Activation from Blood Transcriptome and dsRNA Expression Analyses

**DOI:** 10.3390/ijms27062771

**Published:** 2026-03-18

**Authors:** Ekaterina Spektor, Daniil Poberezhniy, Mikhail Ivanov, Elena Zelenova, Aleksandra Mamchur, Lorena Matkava, Antonina Rumyantseva, Elena Loshakova, Sergey Mitrofanov, Sergey Kucher, Vasilisa Petrova, Lilit Maytesyan, Marina Bocharova, Irina Strazhesko, Olga Tkacheva, Vladimir Yudin, Anton Keskinov, Veronika Skvortsova, Sergey Yudin, Daria Kashtanova

**Affiliations:** 1 Federal State Budgetary Institution “Centre for Strategic Planning and Management of Biomedical Health Risks”, Federal Medical and Biological Agency, Moscow 119121, Russia; 2 Russian Clinical Research Center for Gerontology, Pirogov Russian National Research Medical University, Ministry of Healthcare of the Russian Federation, Moscow 129226, Russia; 3 Federal Medical Biological Agency (FMBA of Russia), Moscow 123182, Russia

**Keywords:** older adults, sleep disorders, sleep maintenance insomnia, transcriptomics, antiviral response, dsRNA

## Abstract

Aging is associated with a high prevalence of insomnia, which is linked to somatic and neuropsychiatric diseases, as well as metabolic and immunological dysfunction. This study aims to identify alterations in the transcriptome profiles and functional metabolic pathways in older adults with different types of sleep disorders. This cross-sectional study included 1002 participants (60–90 years) who were screened for sleep disorders using the Pittsburgh Sleep Quality Index (PSQI) questionnaire. Two types of sleep disorders were identified in the study cohort, i.e., sleep onset insomnia and sleep maintenance insomnia. Both types of insomnia were further analyzed for associations with clinical characteristics, laboratory testing results, and socioeconomic backgrounds. The transcriptomic profiles of peripheral blood samples were examined in 236 individuals, supplemented with differential gene and dsRNA expression analyses (DESeq2). Both sleep onset insomnia and middle insomnia were associated with depression, chronic pain syndrome, and osteoarthritis, while only middle insomnia was associated with cardiometabolic diseases. No associations were observed between sleep onset insomnia or reduced sleep duration and transcriptomic profiles. In contrast, 244 genes were differentially expressed in patients with middle insomnia, indicating the activation of pathways related to viral infection response and inhibition of protein synthesis. Additionally, differential expression analysis of double-stranded RNA (dsRNA) identified 2139 significant changes. Middle insomnia in older adults is associated with transcriptomic changes indicative of an activated antiviral immune response, likely resulting from changes in dsRNA expression levels. The chronic inflammation arising from these transcriptomic alterations may underlie the observed association between middle insomnia and cardiometabolic conditions.

## 1. Introduction

An aging body undergoes numerous cellular, molecular, and physiological changes, including altered sleep patterns [1]. Insomnia, or trouble falling and/or staying asleep, is a common complaint among older people: it affects between 30% and 48% of this demographic [2,3] and 3.9–22.1% of the general population [4]. Substantial evidence indicates a bidirectional relationship between poor sleep quality and the risk of developing somatic diseases. Katz et al. [5] reported that chronic cardiac failure (CCF), chronic obstructive pulmonary disease (COPD), dorsalgia, coxarthrosis, gastric ulcer, angina pectoris, myocardial infarction, osteoarthritis, and prostate diseases pose the highest risk of insomnia. A similar study demonstrated a high prevalence of insomnia in people with arterial hypertension, respiratory and urinary tract diseases, chronic pain, and gastrointestinal diseases.

There is also a reverse correlation between insomnia and the risk of developing somatic and neuropsychiatric disorders. Insomnia has been linked to depression [6], type 2 diabetes mellitus [7], an increased severity of neurodegenerative diseases [8], cardiovascular diseases, and mortality related to cardiovascular conditions [9]. There is limited research regarding the pathophysiological changes underlying sleep disorders, which may offer valuable insights into the relationship between these disorders and internal organ diseases. Recent research has provided accumulating evidence of the transcriptomic alterations associated with insomnia, enhancing our understanding of the molecular mechanisms involved. S. Mithani et al. [10] conducted comprehensive peripheral blood RNA sequencing and identified 288 differentially expressed genes, which are linked to inflammation, ubiquitin system function, and oxidative stress. Functional analysis indicated the involvement of pathways that play a role in the development of metabolic diseases, immune response, and mitochondrial function [11]. In a study by J. Gill et al. [11] involving military personnel, 44 genes were differentially expressed, many of which are known to be associated with the regulation of the sleep–wake cycle (e.g., urotensin 2 and CHPT1), cardiometabolic disease development (e.g., KCNQ1), and ubiquitin system activity.

A number of studies have investigated how gene expression is affected by reduced sleep duration due to deprivation and/or obstructive sleep apnea (OSA) syndrome on gene expression. They have utilized a variety of biological samples, including hair follicles [12], postmortem brain tissue [13], visceral adipose tissue [13], and blood [13]. Gharib et al. [14] showed that OSA activates genes in the NF-κB and the ubiquitination pathways and suppresses the expression of genes encoding the PPAR receptor in adipose tissue. Studies on the effects of sleep deprivation have revealed changes in the transcription of genes involved in immune responses, inflammation, chromatin modification, and stress response.

This study aimed to identify alterations in the transcriptome profiles and functional metabolic pathways in older adults with different types of sleep disorders, as well as to examine the associated comorbidities.

## 2. Results

### 2.1. Cohort Characteristics

The PSQI assessment indicated that 96 participants (9.6%) experienced clinically significant sleep onset insomnia, while 122 participants (12.2%) reported middle insomnia. Additionally, 12 participants (1.2%) experienced both sleep onset and middle insomnia. Table 1 presents the general sleep characteristics of the study cohort. There were differences between men and women in sleep latency and the prevalence of sleep onset insomnia, as well as in the overall PSQI scores, with women exhibiting lower scores.

**Figure 1:**
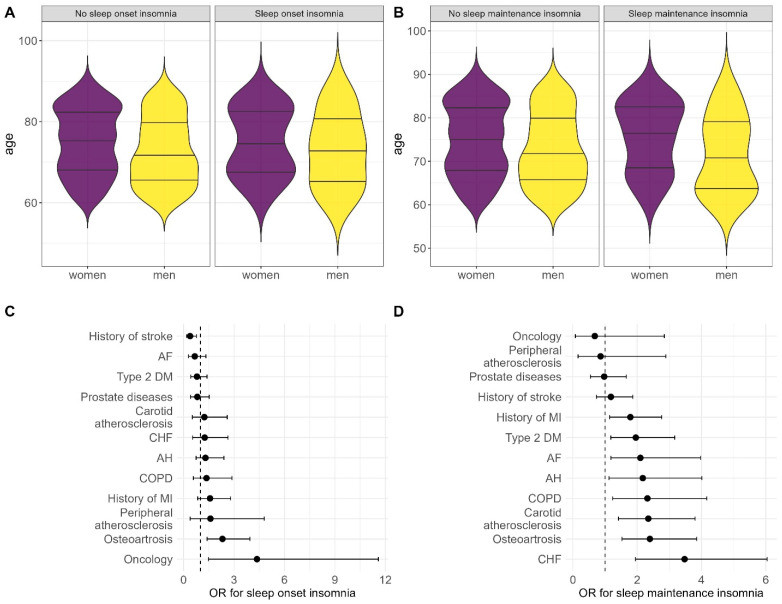
(**A**) Sex and age distribution in patients with sleep onset insomnia and the controls. (**B**) Sex and age distribution in patients with middle insomnia and the controls. (**C**) Odds ratio (OR) and 95% CI for the association between the examined diseases and sleep onset insomnia. (**D**) Odds ratio (OR) and 95% CI for the association between the examined diseases and middle insomnia. PA: peripheral artery; MI: myocardial infarction; COPD: chronic obstructive pulmonary disease; CHF: chronic heart failure; HT: hypertension; DM: diabetes mellitus; AF: atrial fibrillation; ACE: acute cerebrovascular event.

The Supplementary Materials provide comprehensive data on the patients’ clinical characteristics and socioeconomic backgrounds. Table S1 compares patients with and without sleep onset insomnia, while Table S2 compares those with and without middle insomnia. Figure 1A,B displays the participants’ sex and age distributions. The group of patients with sleep onset insomnia was predominantly female (75% vs. 63%; *p* = 0.018). This group was less physically active (5.2% vs. 18%; *p* = 0.003), had higher educational attainment (52% vs. 64%; *p* = 0.027), and had 3.8 times more individuals with low lifelong income (7.3% vs. 1.9%; *p* = 0.003). The prevalence of frailty and sarcopenia was approximately 50% in patients with sleep onset insomnia and 41% and 32% (*p* = 0.023; *p* = 0.007), respectively, in patients without sleep disorders. Notably, the sleep disorder group demonstrated a high prevalence of chronic pain syndrome (76% vs. 46%; *p* < 0.001) and affective disorders (57% vs. 44%; *p* = 0.012), which may trigger or contribute to sleep disorders. No correlation was detected between sleep onset insomnia and personality traits, indicators of cognitive status, marital status, type of employment throughout life, smoking, or alcohol consumption.

Patients with middle insomnia exhibited largely similar characteristics, despite showing minimal similarities with other patients with sleep disorders. This group also had a high proportion of individuals with sarcopenia (52% vs. 31%; *p* < 0.001), frailty (61% vs. 39%; *p* <0.001), chronic pain (74% vs. 45%; *p* < 0.001), and depression (65% vs. 43%; *p* <0.001). Income and educational attainment did not have significance in this group; however, more patients with middle insomnia were single (38% vs. 48%; *p* = 0.035), took daily walks less frequently (49% vs. 72%; *p* < 0.001), were more introverted (an extraversion subscale score of 9 [5; 12] points vs. 11 [9; 13] points; *p* = 0.044) and neurotic (a score of 15 [10; 20] points vs. 9 [7;12] points; *p* = 0.008) according to the Eysenck personality questionnaire, and had higher BMI scores (30.5 kg/m^2^ [26.8; 35.3] vs. 27.5 [24.6; 30.9] kg/m^2^; *p* < 0.001) and higher systolic blood pressure (139 [130; 150] vs. 135 [126, 145 mmHg; *p* = 0.013) and lower FAB scores (16 [14; 17] points vs. 17 [15; 18] points; *p* < 0.001). No correlation was detected between middle insomnia and sex, smoking status, or alcohol use.

### 2.2. Concomitant Diseases of Internal Organs and Laboratory Indicators

Only osteoarthritis and present cancer, as well as acute cerebrovascular events (ACEs), were significantly associated with sleep onset insomnia (Table 2, Figure 1C). Notably, sleep onset insomnia was slightly less prevalent among patients with a history of ACEs. Conversely, middle insomnia was associated with a broader range of comorbidities (Figure 1D), including more frequent nocturnal awakenings observed in patients with chronic heart failure, osteoarthritis, type 2 diabetes mellitus, brachiocephalic artery atherosclerosis, and COPD. The high prevalence of sleep disorders among patients with osteoarthritis appears to be closely related to pain; of 485 patients with chronic pain, 426 (87.8%) had osteoarthritis, suggesting that it may be a primary contributor to the pain syndromes.

No differences in complete blood count and the white blood cell differential count or biochemical blood test, including a lipid profile and hormonal status, as well as individual laboratory parameters, such as C-reactive protein, cortisol in saliva and blood, fibrinogen, D-dimer, leptin, and adiponectin, were observed between patients with sleep onset insomnia (Table S3) and patients with middle insomnia (Table S4).

**Figure 2:**
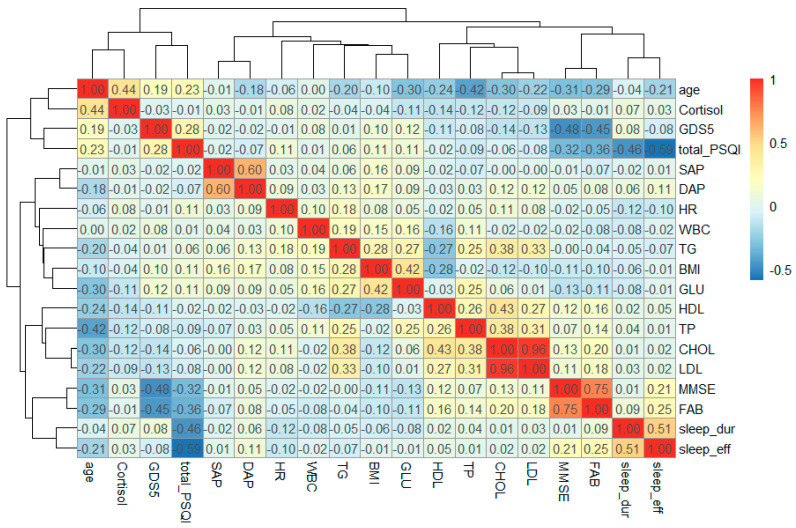
Hierarchically clustered heatmap of continuous clinical variables and selected laboratory parameters. SAP—systolic blood pressure, DAP—diastolic blood pressure, HR—heart rate, BMI—body mass index, total_PSQI—total PSQI score, sleep_dur—sleep duration, sleep_eff—sleep efficiency, GLU—glucose, TP—total protein, TG—triglyceride, CHOL—cholesterol, HDL—high density lipoprotein cholesterol, LDL—low density lipoprotein cholesterol, WBC—white blood cell, Cortisol—urinary cortisol.

Correlation analysis (Figure 2) revealed several significant correlations of at least moderate strength (|ρ| > 0.3) between sleep parameters and other variables. Specifically, the PSQI score was positively correlated with emotional well-being (GDS5 score: ρ = 0.28, *p* < 2 × 10^−16^) and negatively correlated with cognitive performance (MMSE score: ρ = −0.32, *p* < 2 × 10^−16^; FAB score: ρ = −0.36, *p* < 2 × 10^−16^). Sleep efficiency and sleep duration had only weak associations, including a negative correlation between sleep duration and heart rate (ρ = −0.12, *p* = 9.6 × 10^−5^), a negative correlation between sleep efficiency and age (ρ = −0.21, *p* = 5.7 × 10^−9^) and heart rate (ρ = −0.1, *p* = 0.002), as well as positive correlations of sleep efficiency with diastolic blood pressure (ρ = 0.11, *p* = 0.001) and cognitive performance (MMSE score: ρ = 0.21, *p* = 3.6 × 10^−11^; FAB score: ρ = 0.25, *p* = 3.1 × 10^−16^). Notably, the global PSQI score demonstrated stronger associations than its individual components, such as sleep efficiency or sleep duration. However, no significant associations were found between sleep parameters and laboratory markers of metabolic dysfunction. Overall, this analysis reveals a strong interconnection between sleep disturbances, cognitive impairment, and depressive symptoms in our cohort.

### 2.3. Differential Gene Expression Analysis

**Figure 3:**
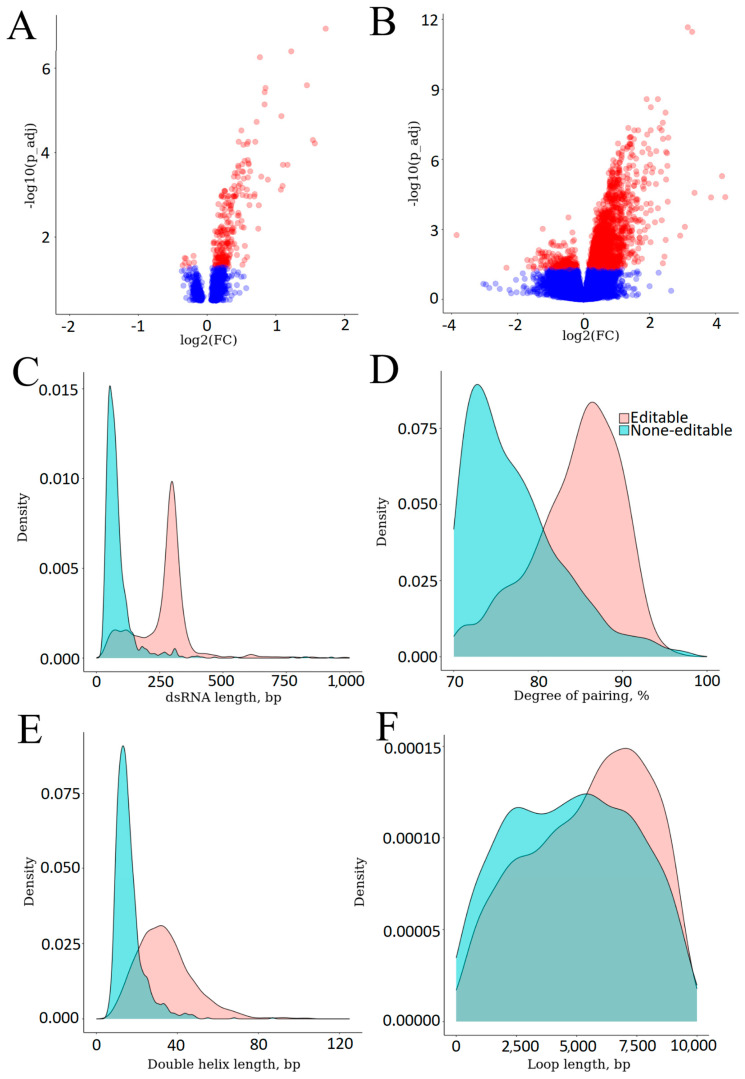
(**A**) Volcano plot showing changes in the expression levels of the examined genes. The red points represent significant differentially expressed genes (*p* adj < 0.05). (**B**) Volcano plot showing changes in dsRNA expression levels. (**C**) Lengths of differentially expressed dsRNA. The red points represent significant differentially expressed genes (*p* adj < 0.05). (**D**) Degrees of pairing of double-stranded regions of differentially expressed dsRNA. (**E**) Lengths of the longest double helices in double-stranded regions of differentially expressed dsRNA. (**F**) Loop lengths in differentially expressed dsRNAP. Bp: base pair.

Total RNA transcriptomic analysis was conducted in a subsample of 236 participants with a BMI below 30 kg/m^2^ to account for this confounder due to the differences observed in the comparative analysis of clinical characteristics. This subsample included 20 patients with sleep onset insomnia and 26 patients with middle insomnia. No statistically significant differences in gene expression levels were found between participants with and without sleep onset insomnia. However, 244 genes exhibited significant differential expression in participants with middle insomnia, with 236 genes being hyperexpressed and only eight genes being hypoexpressed (Figure 3A). Table S5 presents a comprehensive list of differentially expressed genes. To investigate whether the observed associations could be attributed to a reduction in total sleep duration, differential gene expression analysis was conducted comparing a group of patients who slept less than 6 h per day (n = 10) with the control group. No differentially expressed genes were identified.

The dsRNA expression analysis was conducted in 109 patients, including 17 patients with middle insomnia. The analysis revealed 2139 molecules that showed significant changes in expression levels (Figure 3B): 1917 molecules exhibited increased expression levels, while 222 molecules exhibited decreased expression levels. A total of 1165 (54.5%) dsRNAs carried RNA editing sites targeted by the adenosine deaminases acting on RNA (ADAR) enzymes. Figure 3C–F shows the lengths and degrees of pairing of double-stranded regions, as well as the lengths of the longest double-stranded helices and loops in differentially expressed dsRNA.

### 2.4. Functional Analysis

**Figure 4:**
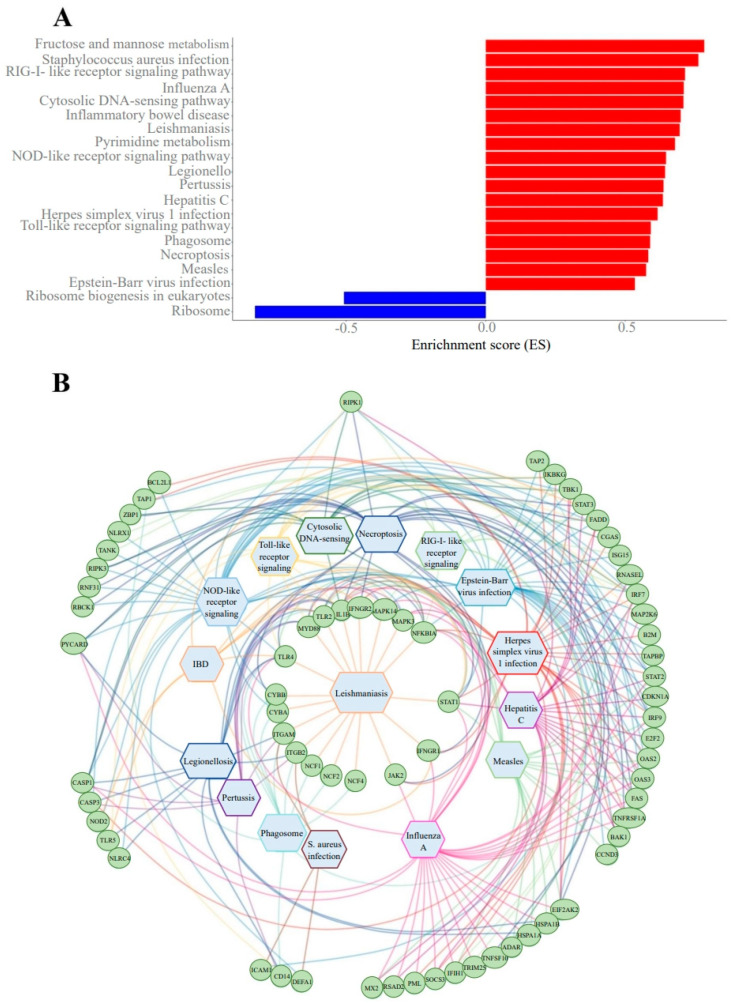
(**A**) Dysregulated functional pathways identified in the GSEA. Red bars represent activated pathways, blue bars represent inhibited pathways. (**B**) Gene–pathway network demonstrating a close relationship between dysregulated pathways mediating the cell’s immune response through the same differentially expressed genes.

Over-representation analysis (ORA) and gene set enrichment analysis (GSEA) were carried out to functionally characterize differentially expressed genes and identify the functional pathways from the KEGG database, in which they are involved. ORA identified 13 enriched functional pathways (Table 3), while GSEA (Figure 4) revealed 20 dysregulated functional pathways, with 18 pathways being activated and two pathways being suppressed. ORA and GSEA produced largely similar results. In both analyses, enriched functional pathways were the ones involved primarily in inflammatory and immune processes, particularly cellular responses to bacterial and viral infections (Table 4).

## 3. Discussion

This study’s findings demonstrate that various types of sleep disorders exhibit distinct clinical characteristics and molecular profiles, as evidenced by transcriptomic alterations. Additionally, the indicators of metabolic imbalance and cardiovascular stress observed in individuals with persistent middle insomnia, along with increased activity in pathways and associated proteins involved in cellular immune responses, align with the current understanding of the strong connection between sleep and autonomic function.

Experiments with sleep deprivation in healthy volunteers demonstrate an increase in the daily secretion of IL-6, TNF-α, and IL-1β [16,17]. Elevated levels of pro-inflammatory markers are generally considered a non-specific body response to stress, tissue damage, or infection. A sharp increase in pro-inflammatory cytokine levels can be a physiological adaptive response; however, sustained elevation may increase the risk of cardiometabolic conditions. Insufficient sleep duration and quality of sleep activate the hypothalamic–pituitary–adrenal (HPA) axis, leading to elevated cortisol levels. This physiological response may influence feeding behaviors, resulting in increased intake of high-calorie foods and accumulation of visceral adipose tissue [18,19]. Visceral obesity is characterized by persistent low-grade inflammation, resulting from elevated levels of pro-inflammatory and reduced levels of anti-inflammatory cytokines [19]. Given its impact on metabolic health, visceral obesity is associated with an increased risk of cardiovascular diseases [20]. This relationship is evidenced by the observed correlations between middle insomnia and increased BMI, as well as hypertension, type 2 diabetes, atrial fibrillation, chronic heart failure, and carotid artery atherosclerosis.

A noteworthy finding of this study is the elevated differential expression of genes that encode proteins involved in the cellular antiviral immune response, even in the absence of clinically significant infections. Table 4 provides a summary of a number of genes that showed the most substantial increase in expression. These genes encode proteins that play a role in viral response, such as cytoplasmic sensors of viral genetic material (such as DNA or double-stranded RNA) and interferon-induced or interferon-inducing proteins. The results of the functional analysis support this observation: the majority of the enriched pathways activated in patients with middle insomnia are involved in the cellular responses to various viral infections. Additionally, the lack of transcriptomic changes in short sleepers indicates that a reduced sleep duration, as well as sleep onset insomnia, in older adults may be less harmful than sleep fragmentation. The natural decline in sleep needs with age helps older adults to better tolerate shortened sleep duration, whereas sleep fragmentation—including loss of deep sleep and REM stages—may lead to disrupted molecular processes. These findings align with existing data on the importance of sleep architecture in maintaining immune homeostasis [16].

The activation of antiviral cellular mechanisms in the absence of obvious reasons in participants with middle insomnia led us to an assumption that endogenous dsRNAs may be involved in the activation of associated metabolic pathways. Research has shown that the increased expression of endogenous dsRNAs due to dysregulation of a range of cellular processes may result in the activation of certain antiviral mechanisms within the cell. Recognized by intracellular dsRNA sensors, like viral dsRNAs, endogenous dsRNAs can trigger innate immune responses, which induce antiviral and inflammatory signaling pathways. To date, several groups of sensory proteins are known to interact with dsRNAs via various mechanisms, with subsequent activation of immunostimulating signaling pathways: RIG-I-like receptors (RLRs), toll-like receptors (TLR), protein kinase R, oligoadenylate synthases (OASes), and NLRP1 (NOD-, LRR- and pyrin domain-containing 1). G. Chen et al. [21] provide a detailed description of the functioning of these dsRNA sensors. The differential gene expression analysis in patients with middle insomnia showed a significant increase in the expression levels of the TLR genes, RIGI and IFIH1 genes encoding RLRs RIG-I and MDA5, respectively, the protein kinase gene R EIF2AK2, and all known OAS genes, such as OAS1, OAS2, OAS3, and OASL. The differential expression analysis of human genome loci that may be involved in dsRNA encoding also demonstrates significant changes in the dsRNome profiles of patients with sleep fragmentation, manifesting as increased expression of almost two thousand endogenous dsRNAs.

RLRs RIG-I and MDA5 recognize dsRNA, which triggers oligomerization and enhances the signal-inducing properties of both receptors. Signaling induction by RIG-I and MDA5 involves relevant E3 ligases—RIPLET and TRIM65—and leads to the activation of type 1 interferon expression. Interferon regulatory factors IRF3 and IRF7 are transcription factors that directly activate the expression of interferon (IFN) genes [21]. Interestingly, the transcriptomic analysis showed increased expression of the IRF7 gene in patients with sleep disorders. Meanwhile, the E3 ligase genes did not exhibit significant changes in expression levels. However, the available data indicates that oligoadenylate synthetase-like (OASL) protein, which is catalytically inactive, and the gene that encodes it are overexpressed in people with sleep fragmentation. The protein is also known to interact with non-ubiquitinated RIG-I, thereby promoting signal induction [22].

It should be noted that dsRNAs recognized by RLRs must have certain properties. In particular, substrates recognized by MDA5 are characterized by long double-stranded regions of RNA molecules, ranging between 300 and 500 bp, according to various sources [21,23]. However, the presence of single unpaired nucleotides and relatively long single-stranded fragments within the double-stranded region, along with ADAR editing sites, common in endogenous dsRNAs, significantly reduces the likelihood of the molecule recognition by MDA5 and autoimmune response induction. Among the differentially expressed dsRNAs detected, 447 were over 300 bp long, making them potential substrates for MDA5, but only 38 of them exhibited a relatively high degree of pairing (>90%) of the double-stranded fragments, with 37 also showing differences in the presence of ADAR editing sites. Unlike MDA5, RIG-I recognizes significantly shorter dsRNAs (ranging from 22 to 200 bp) that possess a 5′-triphosphate group (5′-ppp), which is primarily found in pre-mRNAs and viral RNAs and modified during normal host RNA maturation. After recognizing the 5′-triphosphate group, the first RIG-I translocates along the dsRNA, thereby freeing the end of the molecule, allowing another RIG-I molecule to bind [21,24]. A total of 104 dsRNAs in our study had uninterrupted double helices of over 22 bp in length, potentially available for interaction with RIG-I. The strongest RIG-I agonists are dsRNAs characterized by the presence of a blunt end [24], which was detected in 30 out of 104 dsRNAs.

The recognition of dsRNA by protein kinase R and oligoadenylate synthases OAS1, OAS2, and OAS3, encoding genes which were overexpressed in patients with sleep disorders, triggers mechanisms that inhibit protein biosynthesis. Interestingly, the only two pathways suppressed in this group of patients were pathways involved in protein biosynthesis. Upon binding to the RNA double helix, protein kinase R dimerizes and phosphorylates the translation initiation factor eIF2, which leads to a shutdown of protein synthesis within the cell [25]. Oligoadenylate synthases are characterized by dsRNA-dependent catalytic activity. By binding to the RNA double helix, oligoadenylate synthases catalyze the formation of ATP dimers, secondary messengers to RNase L, which non-selectively degrades both viral RNA and host RNA [26]. Protein kinase R and oligoadenylate synthases recognize fairly short dsRNAs: protein kinase R, ~30 bp; OAS1, <20 bp; and OAS3, >50 bp [21]. A total of 1192 dsRNAs identified in this study were characterized by the presence of at least one double-stranded region over 20 bp in length.

It is safe to conclude that dsRNAs may play a role in the complex process of metabolic dysregulation associated with sleep disorders. Many of the differentially expressed dsRNAs identified in our study possess structural features suggestive of immunostimulatory capacity, realized via the activation of specific cytoplasmic dsRNA receptors. It can be hypothesized that the elevated expression levels of endogenous dsRNAs, combined with disrupted post-transcriptional RNA modification systems that normally regulate their immunostimulatory capacity, may contribute to the activation of cellular immune responses often observed in individuals with sleep disorders. However, the data obtained do not allow us to conclusively determine whether dsRNA destabilization is a contributing factor or a consequence of sleep disorders. Therefore, further experimental validation is necessary.

Several limitations of this study should be acknowledged. First, the relatively small sample size may limit the statistical power and generalizability of our findings. While our cohort size is consistent with several previous differential expression studies in the field [10,11], larger cohorts are necessary to validate these preliminary results. Second, the cross-sectional design does not allow us to make any conclusions about causality, and the possibility of residual confounding cannot be entirely excluded. While we propose a mechanistic model in which endogenous dsRNAs activate antiviral responses leading to chronic inflammation, we acknowledge the lack of direct experimental evidence to support this claim.

This study did not factor in obstructive sleep apnea (OSA), which constitutes one of its limitations. Participants with middle insomnia often experience OSA, which could have contributed to the changes observed. However, they could be considered as non-specific consequences of sleep fragmentation and partial sleep deprivation of any nature. The absence of clinical verification of insomnia diagnoses and the lack of instrumental assessments may be considered another limitation of this study, which relied primarily on the PSQI questionnaire. Nevertheless, the examination of individual types of sleep disorders allowed for the identification of the most adverse sleep disorder profiles, leading to a range of interconnected outcomes. The findings of this study enhance our understanding of the role of sleep. Moreover, insights into the consequences of sleep disorders and their underlying mechanisms may inform modifications to therapeutic strategies in clinical somnology and improve clinical trial practices for sleep medications that not only improve sleep macrostructure but also address the molecular and biological manifestations of insomnia. Further research, including prospective studies, is necessary to identify reliable biomarkers that could serve as surrogate endpoints and aid in establishing causal relationships.

## 4. Materials and Methods

### 4.1. Participant Recruitment

This study is part of a collaborative initiative focused on identifying factors contributing to healthy longevity jointly conducted by the Centre for Strategic Planning and Management of Biomedical Health Risks (CSP) and the Russian Gerontology Research and Clinical Centre (RGRCC). Participants were recruited from patients at RGRCC between 2023 and 2024, with all individuals providing written informed consent prior to participation. The study was approved by the local ethics committee of RGRCC (Protocol No. 76, dated 12 September 2019).

This study included 1002 individuals who were screened for sleep disorders using the Pittsburgh Sleep Quality Index (PSQI) [27]. The participants were 60 to 90 years of age, without established diagnoses of neurodegenerative diseases, psychiatric disorders, or dementia. All participants underwent a detailed assessment of their current social and living conditions, a standard comprehensive geriatric examination, the Mini-Mental State Examination (MMSE) [28], the Frontal Assessment Battery (FAB) [29], the Geriatric Depression Scale (GSD-5), with depression correlating to a score of 2 and above [30], as well as an evaluation for concomitant somatic diseases. Psychometric assessment was administered to 107 participants using the Eysenck personality questionnaire [31], and the Maddy hardiness test [32]. The components of PSQI [27] were evaluated separately. Sleep onset insomnia was defined as a sleep disturbance whereby an individual could not fall asleep within 30 minutes at least three times a week (PSQI questions 2 and 5a); middle insomnia was determined based on the responses to component 5, ranging from 2 to 3 scores. Sleep duration and sleep efficiency were also evaluated separately, with the latter calculated as the percentage of time spent asleep while in bed.

### 4.2. Statistical Analysis

Statistical data analysis was carried out using the R programming language (version 4.4.1) in RStudio (version 2024.09.1+394, RStudio PBC, USA). Descriptive statistics are reported as medians and interquartile ranges for quantitative variables and the absolute number of observations and corresponding proportions within the sample for categorical variables. A comparative analysis was performed using the Mann–Whitney U test for quantitative variables and either Pearson’s chi-square test or Fisher’s exact test for categorical variables, depending on applicability. Correlation analysis was performed using Spearman’s rank correlation coefficient, implemented in base R, with the correlation matrix visualized using the “pheatmap” package, version 1.0.13. Linear regression analysis was conducted to examine linear relationships between continuous variables. Multiple logistic regression was used to evaluate the strength of associations between predictors and various types of sleep disorders. To examine the relationships between diseases and sleep disorders, Fisher’s exact test was employed, and odds ratios with 95% confidence intervals were calculated. The null hypothesis was rejected at *p* < 0.05.

### 4.3. Laboratory Testing, RNA Sequencing and Bioinformatic Analysis of Transcriptomic Data

Blood samples were collected from all participants for clinical and biochemical testing, including coagulation profile and hormonal status. A random subsample of participants was selected for transcriptomic profiling. No additional clinical or demographic selection criteria were applied at this step, ensuring that the subsample remained representative. The Tempus Spin RNA Isolation Kit (Thermo Fisher Scientific, Waltham, MA, USA) was used for total RNA extraction from blood samples collected in Tempus Blood RNA Tubes (Thermo Fisher Scientific, Waltham, MA, USA), with an RNA stabilizer (Thermo Fisher Scientific, Waltham, MA, USA), following the manufacturer’s instructions.

The Illumina TruSeq Stranded Total RNA reagent kit and TruSeq RNA UD Indexes were used for transcriptome sample preparation. The Qubit 4 Fluorometer (Thermo Fisher Scientific, Waltham, MA, USA) was used for library quantification. The Agilent HS D1000 ScreenTape Reagents and the 4200 TapeStation (Agilent Technologies, Santa Clara, CA, USA) were used for library sizing. The NovaSeq 6000 was used for whole-genome sequencing with the S4 Reagent Kit (200 cycles) (Illumina, Way, San Diego, CA, USA) for 2 × 100 bp paired-end reads.

Sequencing quality was assessed using fastQC v. 0.11.9 and MultiQC v. 1.13. Samples containing less than 5 million reads assigned to protein-coding sequences were excluded from the analysis. Salmon v. 1.10.1 [33] was used for quantification of gene expression levels. Human genome assembly GRCh38.p13 and annotations from Gencode v. 46 were used as the reference. To assess expression levels of double-stranded RNA (dsRNA), total RNA reads were trimmed using fastp v. 0.24.0 [34] and aligned to the GRCh38.p14 human genome assembly from Gencode v. 46 using HISAT2 v. 2.2.1 [35]. HTSeq v. 2.0.5 was used for read count with “-a 20”, i.e., MAPQ ≥ 20, and “--mode intersection-strict” settings using human dsRNAome annotations by Andrews et al. [23].

For each phenotypic comparison (e.g., sleep onset insomnia vs. controls), differential expression was assessed using the DESeq2 v. 1.39.8 software package [36]. The Benjamini–Hochberg procedure was used to control the false discovery rate (FDR), with an adjusted *p*-value threshold of <0.05 defining statistical significance. In each group, genes with non-zero expression levels in less than 80% of the total number of samples were excluded from the analysis. No batch effect correction was performed prior to the analysis; however, the batch number, i.e., the cell number, was input as a covariate.

Functional analysis of differentially expressed genes was carried out via over-representation analysis (ORA) and gene set enrichment analysis (GSEA) using the GSEApy v. 1.0.4 and KEGG database. Expressed genome was used as statistical background for ORA. GSEA was performed using the full list of expressed genes ranked based on the log_2_FC value, i.e., log with base 2, or fold change in gene expression levels in the two sample groups.

## Figures and Tables

**Table 1:** Participants’ sleep characteristics.

Characteristic	Entire Cohort, n = 1002	Women, n = 641	Men, n = 361	*p*-Value
Sleep latency, min; Me [Q1, Q3]	15.0 [10.0; 3.0]	15.0 [10.0; 30,0]	10.0 [10.0; 20.0]	0.023
Sleep onset insomnia, n (%)	96 (9.6%)	72 (11%)	24 (6.6%)	0.018
Middle insomnia, n (%)	122 (12%)	87 (14%)	35 (9.7%)	0.074
Sleep duration, hours, Me [Q1, Q3]	7.0 [6,0; 8,0]	7.0 [6,0; 8,0]	7.0 [6.0; 8.0]	0.7
Sleep efficiency, %, Me [Q1, Q3]	88.9 [85,7; 94,7]	88.9 [83,3; 94,7]	88.9 [85.7; 96.0]	0.11
Total PSQI score, Me [Q1, Q3]	5.0 [4,0; 7,0]	6.0 [4.0; 8.0]	5.0 [4.0; 7.0]	<0.001

**Table 2:** Prevalence of sleep disorders in the presence of the examined somatic diseases.

Disease	n	Prevalence of Sleep Onset Insomnia (95% CI)	*p*-Value for Prevalence Difference Compared to the Entire Cohort	Prevalence of Middle Insomnia (95% CI)	*p*-Value for Prevalence Difference Compared to the Entire Cohort
Entire cohort	1002	9.6%	-	12%	-
Hypertension	787	10% (7.9–12.1%)	0.81	13.6% (11.2–16%)	0.01
Type 2 diabetes mellitus	210	8.1% (4.41–11.78%)	0.59	17.7% (12.54–22.87%)	0.001
Atrial fibrillation	145	6.9% (2.77–11.02%)	0.37	19.44% (13–25.89%)	<0.001
History of myocardial infarction	126	13.49% (7.53–19.46%)	0.22	15.08% (8.83–21.33%)	0.077
Chronic heart failure	78	11.54% (4.45–18.63%)	0.72	29.49% (19.37–39.61%)	<0.001
History of stroke	220	4.55% (1.79–7.3%)	0.023	13.64% (9.1–18.17%)	0.095
Atherosclerosis	79	11.39% (4.39–18.4%)	0.74	22.78% (13.54–32.03%)	<0.001
Peripheral artery disease	28	14.29% (1.32–27.25%)	0.61	10.71% (−0.74–22.17%)	>0.99
COPD	73	12.33% (4.79–19.87%)	0.58	21.92% (12.43–31.41%)	0.002
Present cancer	23	30.43% (11.63–49.24%)	0.003	8.7% (−2.82–20.21%)	>0.99
Osteoarthritis	597	12.23% (9.6–14.86%)	0.11	15.6% (12.69–18.52%)	<0.001

**Table 3:** KEGG functional pathways with enriched genes identified in ORA.

Pathway	Adjusted *p*-Value	Differentially Expressed Genes
Influenza A	2.46 × 10^−8^	*RSAD2*, *OAS3*, *IFIH1*, *TNFSF10*, *IRF7*, *OAS2*, *STAT2*, *EIF2AK2*, *PML*, *STAT1*, *MX2*, *CASP1*, *HSPA1A*, *TLR4*, *FAS*, *JAK2*, *ADAR*
Measles	2.46 × 10^−8^	*OAS3*, *IFIH1*, *IRF7*, *OAS2*, *STAT2*, *EIF2AK2*, *STAT1*, *MX2*, *HSPA1A*, *TLR4*, *FAS*, *ADAR*, *CCND3*
NOD-like receptor signaling pathway	2.51 × 10^−6^	*OAS3*, *IRF7*, *OAS2*, *STAT2*, *GBP5*, *GBP3*, *STAT1*, *GBP2*, *NOD2*, *CASP1*, *TLR4*, *RBCK1*, *NLRC4*, *GSDMD*, *TXNIP*, *RNF31*
Necroptosis	1.13 × 10^−5^	*TNFSF10*, *ZBP1*, *STAT2*, *EIF2AK2*, *STAT1*, *CHMP5*, *CASP1*, *TLR4*, *MLKL*, *RBCK1*, *FAS*, *JAK2*, *PARP4*, *RNF31*
Epstein–Barr virus infection	2.56 × 10^−5^	*ISG15*, *OAS3*, *IRF7*, *OAS2*, *STAT2*, *EIF2AK2*, *STAT1*, *TAP1*, *GADD45B*, *TAP2*, *FAS*, *CCND3*
Human immunodeficiency virus 1 infection	6.53 × 10^−4^	*BST2*, *GNB4*, *TAP1*, *TAP2*, *CGAS*, *TLR4*, *FAS*, *PTK2B*, *GNAI2*, *CFL1*
Hepatitis C	1.03 × 10^−4^	*RSAD2*, *IFIT1*, *OAS3*, *IRF7*, *OAS2*, *STAT2*, *EIF2AK2*, *STAT1*, *MX2*, *FAS*
Hepatitis B	0.002	*IFIH1*, *IRF7*, *STAT2*, *STAT1*, *TLR4*, *FAS*, *JAK2*, *PTK2B*
Proteasome	0.015	*PSME2*, *PSMB8*, *PSMB10*, *PSMA4*, *PSMB3*
Legionellosis	0.030	*CASP1*, *HSPA1A*, *TLR4*, *NLRC4*, *ITGAM*
Herpes simplex virus 1 infection	0.030	*OAS3*, *IFIH1*, *IRF7*, *OAS2*, *STAT2*, *EIF2AK2*, *BST2*, *PML*, *STAT1*, *TAP1*, *TAP2*, *CGAS*, *FAS*, *JAK2*, *SP100*
Cytosolic DNA-sensing pathway	0.034	*ZBP1*, *IRF7*, *CGAS*, *CASP1*, *ADAR*
Leishmaniasis	0.044	*STAT1*, *NCF1*, *TLR4*, *JAK2*, *ITGAM*

**Table 4:** Descriptions of differentially expressed genes involved in cellular immune responses.

Biological Function	Gene	Log_2_FC	*p*_adj	Description
Cytoplasmic sensor of dsRNA	*IFIH1*	0.85	2.95 × 10^−6^	*IFIH1* encodes MDA5 which is an intracellular RIG-I-like sensor of viral RNA that triggers the innate immune response.
	*RIGI* (*DDX58*)	0.84	3.71 × 10^−6^	*RIGI* encodes RIG-I protein which is an intracellular receptor of viral RNA, activating the production of type I interferons.
	*DDX60*	0.62	1.75 × 10^−3^	*DDX60* encodes a protein that promotes RIG-I-like receptor-mediated signaling.
	*DDX60L*	0.52	6.81 × 10^−4^	Paralog of DDX60.
	*EIF2AK2*	0.50	4.12 × 10^−3^	*EIF2AK2* encodes an interferon-induced dsRNA-dependent protein kinase R.
	*OAS1*	0.75	1.77 × 10^−3^	Genes with interferon-induced expression encoding dsRNA-dependent 2–5 oligoadenylate synthetases.
	*OAS2*	0.69	1.09 × 10^−3^	
	*OAS3*	1.10	6.17 × 10^−4^	
	*OASL*	1.08	1.37 × 10^−5^	*OASL* is a catalytically inactive 2′–5′-oligoadenylate synthetase, regulating anti-inflammatory signaling through the RIG-I-dependent pathway.
Interferon-inducing genes	*IRF7*	0.71	5.54 × 10^−5^	IRF7 encodes a transcription factor activating the expression of number of virus-induced genes.
	*ZBP1*	0.72	1.88 × 10^−5^	*ZBP1* encodes an interferon-inducing Z-DNA binding protein 1 that acts as an innate immune sensor for foreign DNA, and induces type I interferons production.
Protein-modifying nitrogenous bases in dsRNAs	*ADAR*	0.22	0.026	*ADAR* encodes an enzyme that destabilizes dsRNA molecules by site-specific deamination of adenosine.
Interferon-induced proteins	*IFIT1*	1.54	5.06 × 10^−5^	These genes encode interferon-induced proteins involved in cellular antiviral immune responses.
	*IFIT2*	1.22	4.00 × 10^−7^	
	*IFIT3*	1.10	1.95 × 10^−4^	
	*IFIT5*	0.53	5.97 × 10^−3^	
	*IFI6*	1.07	7.56 × 10^−4^	*IFI6* encodes an interferon-induced protein involved in immune responses to various viral infections.
	*IFI35*	0.88	7.25 × 10^−6^	*IFI35* encodes an alpha interferon-induced regulator of cellular immune response.
	*ISG15*	1.73	1.16 × 10^−7^	*ISG15* encodes a ubiquitin-like protein that gets conjugated to target proteins when induced by alpha and beta interferon.
	*RSAD2*	1.57	6.07 × 10^−5^	*RSAD2* encodes an interferon-induced protein involved in antiviral immune response.
	*MX1*	1.17	1.95 × 10^−4^	*MX1* encodes an interferon type 1- and 2-induced protein involved in cellular immune response.
	*CMPK2*	0.88	4.37 × 10^−4^	*CMPK2* encodes nucleotide monophosphate kinases that act as mediators in immunomodulating processes, including interferon-induced processes, and cellular antiviral immune response.

Log_2_FC: Log_2_ fold change in expression levels using base 2; *p*_adj: adjusted *p*-value. Gene descriptions have been compiled using the GeneCards database [15].

## Data Availability

The data presented in this study are available on request from the corresponding author due to institutional policies and ethics committee restrictions.

## Supplementary Materials

The following supporting information can be downloaded at: https://www.mdpi.com/article/10.3390/ijms27062771/s1.
