# Supplementary material for: Sleep Maintenance Insomnia in Older Adults: Cardiometabolic Comorbidities and Evidence of Antiviral Pathways Activation from Blood Transcriptome and dsRNA Expression Analyses
Source: Int J Mol Sci. 2026 Mar 18;27(6):2771. doi: 10.3390/ijms27062771 (PMC13027132; doi:10.3390/ijms27062771)
Supplement: Supplementary file 1 [file ijms-27-02771-s001.zip › Tables S1-S4.pdf]

Table S1. Clinical characteristics and socio-economic parameters associated with sleep onset insomnia.

Parameter	Entire cohort, n = 1002	No sleep onset insomnia, n = 906	Sleep onset insomnia, n = 96	p-value
Age, years; Me [Q1; Q3]	74.0 [67.0; 82.0]	74.0 [67.0; 82.0]	73.0 [67.0; 83.0]	0.75
Men, n (%)	361 (36%)	337 (37%)	24 (25%)	**0.018**
BMI, kg/m^2, Me [Q1; Q3]	27.8 (24.9; 31.2)	27.9 (24.9; 31.2)	27.3 (24.7; 30.8)	0.38
Systolic blood pressure, mm Hg; Me [Q1; Q3]	135.0 (127.0; 145.0)	135.0 (127.0; 146.0)	132.0 (120.5; 140.0)	**0.007**
Frailty, n (%)	423 (42%)	372 (41%)	51 (53%)	**0.023**
Sarcopenia, n (%)	335 (33%)	291 (32%)	44 (46%)	**0.007**
Physical activity, n (%)	166 (17%)	161 (18%)	5 (5.2%)	**0.003**
Daily walks, n (%)	697 (70%)	627 (69%)	70 (73%)	0.53
Married, n (%)	471 (47%)	431 (48%)	40 (42%)	0.34
Graduate degree, n (%)	631 (63%)	581 (64%)	50 (52%)	**0.027**
Currently employed, n (%)	162 (16%)	149 (16%)	13 (14%)	0.46
Primarily lifelong mental work, n (%)	672 (67%)	613 (68%)	59 (61%)	0.4
Lifelong low income, n (%)	24 (2.4%)	17 (1.9%)	7 (7.3%)	**0.003**
Religious practice, n (%)	702 (70%)	625 (69%)	77 (80%)	**0.022**
Hobbies, n (%)	761 (76%)	691 (76%)	70 (73%)	0.47
Current smoker, n (%)	82 (8.2%)	76 (8.4%)	6 (6.3%)	0.6
Frequent alcohol consumption (>1 time per week), n (%)	31 (3.1%)	29 (3.2%)	2 (2%)	0.76
Chronic pain, n (%)	485 (48%)	412 (46%)	73 (76%)	**<0.001**
MMSE; Me [Q1, Q3]	28 [23; 29]	28 [23; 29]	28 [23; 29]	0.5
FAB; Me [Q1, Q3]	16.0 [14.0; 18.0]	16.0 [14.0; 18.0]	17.0 [15.0; 18.0]	**0.059**
Maddy’s hardiness test, total score; Me [Q1, Q3]	18.0 [16.0; 23.0]	18.5 [16.0; 23.0]	16.5 [12.0; 21.0]	0.18

The Eysenck Personality Inventory (extraversion), total score; Me [Q1, Q3]	11.0 [9.0; 13.0]	11.0 [9.0; 13.0]	10.0 [9.0; 11.0]	0.2
The Eysenck Personality Inventory (neuroticism), total score; Me [Q1, Q3]	10.0 [7.0; 13.0]	9.0 [7.0; 13.0]	10.0 [5.0; 17.0]	0.86
Depression (GDS-5), n (%)	453 (45%)	398 (44%)	55 (57%)	**0.012**

Table S2. Clinical characteristics and socio-economic parameters associated with middle insomnia.

Parameter	Entire cohort, n = 1001	No nocturnal awakenings, n = 879	Frequent nocturnal awakenings, n = 122	p-value
Age, years; Me [Q1; Q3]	74.0 [67.0; 82.0]	73.0 [67.0; 82.0]	75.5 [67.0; 82.0]	0.8
Men, n (%)	360 (36%)	325 (37%)	35 (29%)	0.074
BMI, kg/m^2, Me [Q1; Q3]	27.8 (24.9; 31.2)	27.5 (24.7; 30.9)	30.6 (26.9; 35.5)	**<0.001**
Systolic blood pressure, mm Hg; Me [Q1; Q3]	135.0 (127.0; 145.0)	135.0 (126.0; 145.0)	140.0 (130.0; 151.0)	**0.001**
Frailty, n (%)	422 (42%)	347 (39%)	75 (61%)	**<0.001**
Sarcopenia, n (%)	334 (33%)	270 (31%)	64 (52%)	**<0.001**
Physical activity, n (%)	165 (16%)	152 (17%)	13 (11%)	0.085
Daily walks, n (%)	696 (70%)	636 (72%)	60 (49%)	**<0.001**
Married, n (%)	470 (47%)	425 (48%)	46 (38%)	**0.035**
Graduate degree, n (%)	630 (63%)	558 (63%)	72 (59%)	0.39
Currently employed, n (%)	162 (16%)	139 (16%)	23 (19%)	0.4
Primarily lifelong mental work, n (%)	671 (67%)	590 (67%)	81 (66%)	0.95
Lifelong long income, n (%)	24 (2.4%)	21 (2.4%)	3 (2.5%)	>0.99
Practicing a religion, n (%)	701 (70%)	612 (70%)	89 (73%)	0.52
Hobbies, n (%)	760 (76%)	667 (76%)	93 (76%)	>0.99
Current smoker, n (%)	82 (8.2%)	69 (7.9%)	13 (11%)	0.38
Frequent alcohol consumption (>1 time per week),	31 (3.1%)	25 (2.9%)	6 (5%)	0.34

n (%)				
Chronic pain, n (%)	484 (48%)	394 (45%)	90 (74%)	**<0.001**
MMSE; Me [Q1; Q3]	28 [23; 29]	28 [23; 29]	28 [23; 29]	0.072
FAB; Me [Q1; Q3]	16.0 [14.0; 18.0]	17.0 [15.0; 18.0]	16.0 [14.0; 17.0]	**<0.001**
Maddy's hardiness test. total score; Me [Q1; Q3]	18.0 [16.0; 23.0]	19.0 [16.0; 23.0]	18.0 [14.0; 21.0]	0.19
The Eysenck Personality Inventory (extraversion), total score; Me [Q1; Q3]	11.0 [9.0; 13.0]	11.0 [9.0; 13.0]	9.0 [5.0; 12.0]	**0.044**
The Eysenck Personality Inventory (neuroticism), total score; Me [Q1; Q3]	10.0 [7.0; 13.0]	9.0 [7.0; 12.0]	15.0 [10.0; 20.0]	**0.008**
Depression (GDS-5), n (%)	453 (45%)	374 (43%)	79 (65%)	**<0.001**

Table S3. Laboratory indicators in patients with and without sleep onset insomnia. All variables are provided in Me [Q1; Q3].

**Indicator**	**Sleep onset insomnia**	**No sleep onset insomnia**	**p-value**	**p_adj**
Hemoglobin, g/dL	13.8 [13.07; 14.7]	13.9 [13.0; 14.8]	0.998775497	0.998775497
Hematocrit, %	40.7 [38.44; 43.86]	41.25 [38.3; 43.7]	0.86838692	0.979244825
Platelets, *10^9/л	218.0 [192.7; 261.6]	234.0 [198.0; 274.0]	0.160122234	0.848647839
Red blood cells, *10^{12}/л	4.67 [4.34; 5.06]	4.62 [4.28; 4.92]	0.312695855	0.941351866
While blood cells, *10^9/л	5.87 [4.85; 7.24]	5.92 [5.0; 6.99]	0.978273556	0.998775497
Red Blood Cell Distribution Width (RDW) %	13.4 [12.9; 14.0]	13.5 [13.0; 14.2]	0.129536544	0,848647839
Mean Corpuscular Hemoglobin (MCH), pg	29.8 [28.87; 30.9]	30.2 [29.2; 31.35]	0.106936539	0.824009733
Mean Corpuscular Hemoglobin Concentration (MCHC), g/dL	33.9 [33.3; 34.33]	33.8 [33.1; 34.5]	0.621647459	0.941351866
Mean Corpuscular Volume (MCV), fl	87.7 [86.08; 90.82]	89.5 [86.7; 92.2]	0.034883822	0.54324305
Erythrocyte Sedimentation Rate (ESR), mm/h	13.0 [6.95; 22.05]	11.0 [6.0; 22.0]	0.732659972	0.94987572
Basophils, %	0.6 [0.4; 0.8]	0.5 [0.3; 0.8]	0.108831474	0.824009733
Absolute basophil count (ABC)., *10^9/L	0.04 [0.02; 0.05]	0.03 [0.02; 0.05]	0.267236707	0.941351866
Eosinophils, %	2.6 [1.77; 4.03]	2.2 [1.5; 3.4]	0.06679007	0.70797474
Absolute eosinophil count (AEC), *10^9/L	0.16 [0.1; 0.23]	0.13 [0.09; 0.2]	0.040999475	0.54324305

Lymphocytes, %	29.3 [23.55; 34.9]	30.0 [25.3; 35.58]	0.470304014	0.941351866
Absolute lymphocyte count (ALC), *10^9/L	1.66 [1.38; 2.23]	1.76 [1.38; 2.15]	0.510650528	0.941351866
Monocytes, %	7.8 [6.4; 8.8]	7.3 [6.1; 8.78]	0.15405655	0.848647839
Absolute monocyte count, *10^9/L	0.45 [0.35; 0.62]	0.43 [0.35; 0.54]	0.251901073	0.941351866
Absolute neutrophil count (ANC), *10^9/L	3.64 [2.64; 4.6]	3.4 [2.77; 4.2]	0.862121114	0.979244825
Neutrophils, %	58.0 [51.88; 65.46]	58.9 [53.42; 64.28]	0.808726041	0.974147277
Absolute lymphocyte count (ALC), *10^9/L	1.63 [1.41; 2.18]	1.77 [1.44; 2.19]	0.285526233	0.941351866
Lymphocytes, %	29.65 [23.0; 35.22]	30.2 [25.04; 35.53]	0.778191241	0.959165948
Total bilirubin, µmol/L	11.5 [8.4; 15.58]	11.2 [8.3; 15.0]	0.752731702	0.94987572
Gamma-glutamyl transferase (GGT), U/L	21.0 [17.0; 36.8]	22.0 [15.0; 30.0]	0.398352032	0.941351866
Glucose, mmol/l	5.5 [5.1; 6.1]	5.5 [5.0; 6.2]	0.897795062	0.991315381
HbA1c, %	5.6 [5.4; 5.8]	5.6 [5.3; 5.83]	0.837180581	0.979244825
Creatinine, µmol/l	79.0 [69.0; 90.6]	80.0 [67.0; 94.05]	0.598951479	0.941351866
Urea, mmol/l	6.3 [5.12; 7.38]	6.0 [4.8; 7.4]	0.728423417	0.94987572
Uric acid, µmol/l	342.0 [287.6; 379.8]	328.0 [282.0; 385.8]	0.53808876	0.941351866
Total protein, g/L	74.0 [71.0; 76.8]	74.0 [70.45; 78.0]	0.608248159	0.941351866
Albumin, g/L	43.3 [40.96; 46.0]	43.7 [41.1; 46.2]	0.747098683	0.94987572
Triglycerides, mmol/L	1.29 [1.05; 1.92]	1.34 [1.01; 1.78]	0.563017544	0.941351866
Cholesterol, mmol/L	5.54 [4.74; 7.15]	5.56 [4.46; 6.49]	0.492077768	0.941351866
HDL cholesterol, mmol/L	1.45 [1.15; 1.74]	1.4 [1.15; 1.67]	0.447564893	0.941351866
LDL cholesterol, mmol/L	3.39 [2.39; 4.64]	3.42 [2.49; 4.22]	0.684310224	0.94987572
Alkaline phosphatase, U/L	82.0 [68.4; 97.8]	84.0 [71.0; 100.0]	0.701368032	0.94987572
Alanine aminotransferase (ALT), U/L	17.0 [13.0; 23.8]	16.0 [12.0; 21.0]	0.294143448	0.941351866
Aspartate Aminotransferase (AST), U/L	21.0 [17.2; 24.0]	20.0 [17.0; 24.0]	0.246258029	0.941351866
Alpha-1-globulins, g/L	2.9 [2.7; 3.2]	2.9 [2.7; 3.2]	0.547468732	0.941351866

Alpha-2 globulins, g/L	7.4 [6.8; 8.28]	7.3 [6.6; 8.1]	0.32701727	0.941351866
Gamma globulins, g/L	10.7 [9.66; 12.5]	11.4 [9.9; 13.31]	0.182068336	0.877238347
Beta-globulins, g/L	8.2 [7.62; 9.1]	8.4 [7.7; 9.1]	0.681235289	0.94987572
Ferritin, µg/L	73.5 [51.0; 126.85]	90.0 [48.2; 149.0]	0.570591985	0.941351866
Free T3, pmol/L	4.2 [3.87; 4.6]	4.2 [3.74; 4.64]	0.943475129	0.998775497
Thyrotropin releasing hormone (TSH), mU/L	2.21 [1.59; 3.3]	1.75 [1.21; 2.59]	0.001195922	0.063383855
25(OH) Vitamin D, ng/mL	26.5 [14.24; 39.68]	20.2 [10.0; 32.78]	0.024762637	0.54324305
Adiponectin, mcg/mL	32.53 [23.07; 50.93]	29.36 [21.3; 47.11]	0.420470918	0.941351866
Leptin, ng/mL	12.16 [5.87; 36.55]	13.85 [5.9; 34.56]	0.996790123	0.998775497
Cortisol, nmol/L	375.2 [278.74; 483.42]	348.0 [274.47; 457.14]	0.497334113	0.941351866
C-reactive protein, mg/L	1.96 [0.7; 3.99]	1.64 [0.77; 3.13]	0.539187852	0.941351866
D-dimer, µg/mL	148.0 [93.0; 226.4]	158.0 [108.0; 240.55]	0.517857398	0.941351866
Fibrinogen, g/L	3.75 [3.4; 3.9]	3.65 [3.3; 4.0]	0.341722924	0.941351866
Cortisol in saliva, ng/mL	4.7 [3.05; 9.83]	4.85 [3.08; 8.75]	0.943703503	0.998775497

Note: indicators are presented as medians and interquartile ranges (Me [Q1; Q3]).

Table S4. Laboratory indicators in patients with and without middle insomnia. All variables are provided in Me [Q1; Q3].

**Indicator**	**Middle insomnia**	**No middle insomnia**	**p-value**	**p_adj**
Hemoglobin, g/dL	13.8 [13.1; 14.93]	13.9 [12.9; 14.8]	0.625232562	0.8956034
Hematocrit, %	40.3 [38.17; 43.79]	41.3 [38.3; 43.7]	0.801066554	0.943478386
Platelets, *109/л	241.0 [199.4; 282.9]	233.0 [197.0; 270.0]	0.402817371	0.737494287
Red blood cells, *1012/л	4.61 [4.28; 4.92]	4.62 [4.29; 4.93]	0.952406663	0.961623475
While blood cells, *109/л	5.79 [5.06; 7.33]	5.92 [4.96; 6.98]	0.907940825	0.961623475
Red Blood Cell Distribution Width (RDW) %	13.4 [13.0; 14.0]	13.5 [13.0; 14.1]	0.283411337	0.686339051
Mean Corpuscular Hemoglobin (MCH), pg	30.4 [29.1; 31.83]	30.1 [29.1; 31.2]	0.145547144	0.578897463

Mean Corpuscular Hemoglobin Concentration (MCHC), g/dL	34.1 [33.57; 34.7]	33.7 [33.1; 34.4]	0.001289085	0.068321518
Mean Corpuscular Volume (MCV), fl	89.2 [86.54; 92.2]	89.3 [86.64; 92.16]	0.927314969	0.961623475
Erythrocyte Sedimentation Rate (ESR), mm/h	14.0 [6.7; 22.6]	11.0 [6.0; 22.0]	0.458039745	0.758628327
Basophils, %	0.6 [0.4; 0.8]	0.5 [0.3; 0.8]	0.499659489	0.762125088
Absolute basophil count (ABC)., *109/L	0.04 [0.02; 0.05]	0.03 [0.02; 0.05]	0.439636133	0.753871791
Eosinophils, %	2.4 [1.8; 3.79]	2.2 [1.5; 3.4]	0.148561561	0.578897463
Absolute eosinophil count (AEC), *109/L	0.15 [0.09; 0.21]	0.13 [0.08; 0.2]	0.163838905	0.578897463
Lymphocytes, %	30.0 [24.81; 34.62]	29.9 [25.12; 35.58]	0.673081047	0.925296663
Absolute lymphocyte count (ALC), *109/L	1.65 [1.37; 2.06]	1.76 [1.39; 2.15]	0.373981736	0.737494287
Monocytes, %	7.9 [6.47; 9.1]	7.3 [6.1; 8.7]	0.157834856	0.578897463
Absolute monocyte count, *109/L	0.46 [0.35; 0.56]	0.43 [0.34; 0.54]	0.355515523	0.737494287
Absolute neutrophil count (ANC), *109/L	3.39 [2.83; 4.28]	3.44 [2.72; 4.22]	0.894061706	0.961623475
Neutrophils, %	57.7 [53.8; 63.49]	58.9 [52.92; 64.6]	0.932318628	0.961623475
Absolute lymphocyte count (ALC), *109/L	1.69 [1.37; 2.1]	1.77 [1.46; 2.19]	0.270853805	0.686339051
Lymphocytes, %	30.0 [23.16; 35.61]	30.2 [25.1; 35.43]	0.40353461	0.737494287
Total bilirubin, µmol/L	12.75 [9.2; 14.91]	11.0 [8.2; 15.0]	0.065746056	0.497791566
Gamma-glutamyl transferase (GGT), U/L	21.0 [16.0; 32.1]	22.0 [15.0; 30.0]	0.517669871	0.762125088
Glucose, mmol/l	5.8 [5.2; 6.3]	5.5 [5.0; 6.1]	0.030979685	0.402915153
HbA1c, %	5.6 [5.4; 5.9]	5.6 [5.3; 5.8]	0.440943878	0.753871791
Creatinine, µmol/l	79.5 [66.95; 93.1]	80.0 [68.0; 94.0]	0.800560993	0.943478386
Urea, mmol/l	6.6 [5.4; 8.0]	5.9 [4.8; 7.3]	0.010383636	0.275166342
Uric acid, µmol/l	341.0 [315.95; 388.05]	325.5 [279.0; 385.0]	0.038010864	0.402915153
Total protein, g/L	74.0 [71.0; 77.0]	74.0 [70.0; 78.0]	0.706528323	0.927111956
Albumin, g/L	43.5 [41.18; 45.9]	43.7 [41.1; 46.28]	0.738129644	0.931449313
Triglycerides, mmol/L	1.38 [1.01; 1.7]	1.34 [1.02; 1.79]	0.921553249	0.961623475

Cholesterol, mmol/L	5.34 [4.38; 6.32]	5.61 [4.49; 6.51]	0.325239973	0.718238274
HDL cholesterol, mmol/L	1.36 [1.17; 1.68]	1.41 [1.14; 1.68]	0.717199815	0.927111956
LDL cholesterol, mmol/L	3.19 [2.33; 4.01]	3.46 [2.51; 4.29]	0.159469674	0.578897463
Alkaline phosphatase, U/L	82.5 [73.9; 99.1]	84.0 [70.0; 100.0]	0.932602516	0.961623475
Alanine aminotransferase (ALT), U/L	16.0 [12.0; 21.0]	16.0 [12.0; 22.0]	0.961623475	0.961623475
Aspartate Aminotransferase (AST), U/L	20.0 [17.0; 23.0]	20.0 [17.0; 24.0]	0.485597171	0.762125088
Alpha-1-globulins, g/L	3.05 [2.7; 3.3]	2.9 [2.7; 3.2]	0.024376831	0.402915153
Alpha-2 globulins, g/L	7.6 [6.9; 8.2]	7.3 [6.6; 8.1]	0.284895455	0.686339051
Gamma globulins, g/L	11.05 [9.99; 12.6]	11.4 [9.9; 13.38]	0.088146392	0.547702964
Beta-globulins, g/L	8.5 [7.9; 9.1]	8.4 [7.6; 9.1]	0.192927194	0.63907133
Ferritin, µg/L	77.0 [41.6; 150.8]	89.0 [50.0; 148.05]	0.379603756	0.737494287
Free T3, pmol/L	4.15 [3.8; 4.6]	4.2 [3.76; 4.63]	0.785560837	0.943478386
Thyrotropin releasing hormone (TSH), mU/L	1.56 [1.01; 2.44]	1.88 [1.29; 2.68]	0.048500349	0.428419748
25(OH) Vitamin D, ng/mL	19.15 [9.8; 30.0]	21.15 [10.32; 34.0]	0.31313237	0.718238274
Adiponectin, mcg/mL	30.42 [24.85; 51.94]	29.41 [20.3; 46.67]	0.093006164	0.547702964
Leptin, ng/mL	15.64 [7.08; 34.74]	13.44 [5.66; 34.76]	0.277037172	0.686339051
Cortisol, nmol/L	360.1 [278.17; 519.29]	346.85 [275.24; 452.72]	0.258744914	0.686339051
C-reactive protein, mg/L	1.92 [0.96; 3.8]	1.64 [0.76; 3.13]	0.242652948	0.686339051
D-dimer, µg/mL	154.0 [101.4; 223.5]	158.0 [105.0; 241.0]	0.680878677	0.925296663
Fibrinogen, g/L	3.7 [3.4; 4.2]	3.6 [3.3; 4.0]	0.127950115	0.578897463
Cortisol in saliva, ng/mL	6.01 [3.45; 10.04]	4.67 [2.99; 8.63]	0.515796486	0.762125088
Note: indicators are presented as medians and interquartile ranges (Me [Q1; Q3]).				
